# Decaying trees improve nesting opportunities for cavity‐nesting birds in temperate and boreal forests: A meta‐analysis and implications for retention forestry

**DOI:** 10.1002/ece3.4245

**Published:** 2018-07-16

**Authors:** Fabian Gutzat, Carsten F. Dormann

**Affiliations:** ^1^ University of Freiburg Freiburg Germany

**Keywords:** cavity nesters, conservation, meta‐analysis, nest‐site selection, retention forestry, tree characteristics

## Abstract

Many studies have dealt with the habitat requirements of cavity‐nesting birds, but there is no meta‐analysis on the subject and individual study results remain vague or contradictory. We conducted a meta‐analysis to increase the available evidence for nest‐site selection of cavity‐nesting birds. Literature was searched in Web of Science and Google Scholar and included studies that provide data on the habitat requirements of cavity‐nesting birds in temperate and boreal forests of varying naturalness. To compare nest and non‐nest‐tree characteristics, the following data were collected from the literature: diameter at breast height (DBH) and its standard deviation (*SD*), sample size of trees with and without active nest, amount of nest and available trees described as dead or with a broken crown, and amount of nest and available trees that were lacking these characteristics. Further collected data included bird species nesting in the cavities and nest‐building type (nonexcavator/excavator), forest type (coniferous/deciduous/mixed), biome (temperate/boreal), and naturalness (managed/natural). From these data, three effect sizes were calculated that describe potential nest trees in terms of DBH, vital status (dead/alive), and crown status (broken/intact). These tree characteristics can be easily recognized by foresters. The results show that on average large‐diameter trees, dead trees, and trees with broken crowns were selected for nesting. The magnitude of this effect varied depending primarily on bird species and the explanatory variables forest type and naturalness. Biome had lowest influence (indicated by ΔAIC). We conclude that diameter at breast height, vitality, and crown status can be used as tree characteristics for the selection of trees that should be retained in selectively harvested forests.

## 1 INTRODUCTION

Avian diversity is essential for the provisioning of forest ecosystem services (e.g., pest control, seed dispersal, or recreational value of a forest: Fayt, Machmer, & Steeger, 2005; Sekercioglu, 2006). A significant part of this avian diversity is made up of cavity‐nesting birds (van der Hoek, Gaona, & Martin, 2017), and their habitat requirements are a recurrent object of study (e.g. Bull, 1987; O'Halloran & Conner, 1987; Conway & Martin, 1993; Steeger & Hitchcock, 1998; Poulin, Villard, Edman, Goulet, & Eriksson, 2008; Tremblay, Savard, & Ibarzabal, 2015; Geleynse, Nol, Burke, & Elliott, 2016). These studies are also of importance for avian biodiversity in general as it has been shown that woodpecker richness indicates overall avian richness across harvesting systems and forest conditions (Drever, Aitken, Norris, & Martin, 2008; Drever & Martin, 2010).

Frequently measured variables to characterize cavity‐nesting bird habitats are diameter at breast height (DBH), tree vital status (dead/alive), and tree crown status (broken/intact) (e.g. Aubry & Raley, 2002; Dornak, Burt, Coble, & Conner, 2004; Martin, Aitken, & Wiebe, 2004; Tremblay et al., 2015). These tree characteristics are easily recognized by foresters and therefore well‐suited for the formulation of forest management recommendations.

However, there is conflicting evidence with regard to DBH as a habitat describing variable. Some studies suggest that trees with a large DBH are selected for nesting (e.g. Tremblay et al., 2015), while others state the opposite or do not come to a clear conclusion (e.g. Gentry & Vierling, 2008; Milne & Hejl, 1989; Schreiber & deCalesta, 1992; Seavy, Burnett, & Taille, 2012). Similarly, there is a lack of explicit evidence about the selected vital status (dead/alive) of a nest tree (e.g. Dobkin, Rich, Pretare, & Pyle, 1995; Hutto & Gallo, 2006; Martin et al., 2004) and its crown status (broken/intact: e.g., Martin et al., 2004; Seavy et al., 2012). Accordingly, the suitability of instantly visible tree characteristics (e.g., broken top) to describe the habitat requirements has been questioned (Lorenz, Vierling, Johnson, & Fischer, 2015). In cases of conflicting evidence, a synthesis of available study results is recommended (CEE, 2013; Koricheva & Gurevitch, 2014). The advantage of meta‐analyses over other techniques (e.g., narrative reviews) used for summarizing study results is the consideration of individual study sample sizes to statistically estimate the magnitude of the underlying effect size (Koricheva, Gurevitch, & Mengersen, 2013, p. 8, table 1.1). Considering that there exists a substantial body of literature, it is surprising that a meta‐analysis of the habitat requirements for cavity‐nesting birds is still lacking.

Besides being of general interest for the conservation of avian biodiversity, cavity‐nesting birds act as keystone species on a variety of grounds. Most obviously, woodpecker cavities benefit numerous subsequent nonexcavating bird species (nonexcavators; e.g., Daily, Ehrlich, & Haddad, 1993; Martin et al., 2004). Daily and Ehrlich (1988) reported that sap wells drilled by red‐naped sapsuckers (*Sphyrapicus nuchalis*) were also used for feeding by other species including warblers, hummingbirds, and chipmunks. These observations illustrate another key role cavity nesters play in ecosystems and underline the neccessity for explicit evidence regarding their habitat requirements.

Considering the importance of cavity nesters as indicator and keystone species, it is more than an academic interest to better understand their ecological requirements. We hypothesized that large trees, trees with broken crowns, and dead trees are selected for nesting by cavity‐nesting birds.

The objectives of this study were to (a) evaluate whether cavity‐nesting birds select for nest trees with large DBH, dead trees, and trees with broken crowns. We also aimed to elucidate the influence of (b) dominating tree species and (c) naturalness in (d) temperate and boreal forests. In conjunction to the use of meta‐analytical methods to quantify nest‐tree selection of cavity‐nesting birds, we also provide an overview of management recommendations extracted from the reviewed studies as a scientific basis to (e) guide future management decisions in the selection of trees that should be retained in temperate and boreal forests.

## 2 METHODS

Studies were included in the analysis according to a priori study inclusion criteria (see Supporting information List S1). To broaden the range of studies, the search strings (see Supporting information Table S1) used were formulated as general as possible and contained various terms commonly used in studies related to cavity‐nesting birds. We searched for literature from any year of publication on cavity‐nesting birds in nontropical forests in the following databases: Google Scholar, Web of Science, CAB Abstracts, GeoRef, BioOne, ScienceDirect, JSTOR, and Springer. All hits found by Web of Science were included for further analysis. Similar to Fedrowitz et al. (2014) the first 100 hits (sorted by relevance) of each of the remaining databases were examined. Indeed, most relevant studies were found within the first 50 hits in all databases. Only between zero and four studies appeared in the second 50 hits indicating that we did not exclude useful studies by restricting ourselves, at this step, to 100 studies. The search was conducted in September 2016 and updated in November 2017 in Web of Science. Reference lists of included studies were searched for further potentially relevant primary studies. Several studies which we could not access via the library or online were sent to us upon request by the authors. We checked studies that published data from the same research area in different years or journals. Such duplicates were excluded. We conducted two rounds of study selection. In the first round, study title and abstract were read and the study excluded if it did not meet our criteria (see Supporting information List S1). In the second round, the studies included in the first round were read in their entirety to assess the fulfillment of our study inclusion criteria. The first round of study selection yielded 453 studies. After the second round, 51 studies remained. Seven studies were found in the references of studies considered relevant for the meta‐analysis. The following data were extracted from the studies:

1. DBH (breast height = approx. 1.3 m above ground) and standard deviation (*SD*) [cm], including the sample size (*N*) of dead and live trees with and without active nests. Most studies provided information to separate nest‐ from non‐nest trees. However, this separation was not always possible. If fewer than 25% of random trees were nest trees, then the study was included (Supporting information Appendix S1: study inclusion criteria 3b). Therefore, available trees were defined as the entire pool of trees containing non‐nest trees and nest trees.
2. Nest height [m].
3. Amount of nest and available trees described as dead (stage 3 trees, Thomas, Anderson, Maser, & Bull, 1979) or with a broken crown. Amount of nest and available trees that were lacking these characteristics. Trees with a broken crown were typically described by the authors as being dead (in line with stage 6 trees, Thomas et al., 1979), but some trees may have had living limbs.
4. Bird species nesting in the cavity and bird type (nonexcavator/excavator). Weak and primary excavators were pooled in the category “excavators” following the example of previous studies (e.g. Li & Martin, 1991; Raphael & White, 1984). Nonexcavators are birds that use excavator cavities (e.g. Raphael & White, 1984). If only one overall value for several bird species was given (e.g., mean nest tree DBH), bird species was assigned the label “unspecified. “
5. Biome (temperate/boreal) and latitude (in decimal degree) based on the study site description and complemented by a map of the world biomes (linked in Supporting information List S1).
6. Forest type based on the dominating tree species (coniferous/deciduous/mixed).
7. Naturalness (managed/natural). Often either forest age or past management history was provided in studies (see also Remm & Lõhmus, 2011). We combined this information to provide a measure of forest stand naturalness. Forests classified as natural were typically mature with large trees, although previous cuttings may have taken place as few forest stands in our data can be considered unmanaged.
8. Quantitative management recommendations.

**Figure 1:**
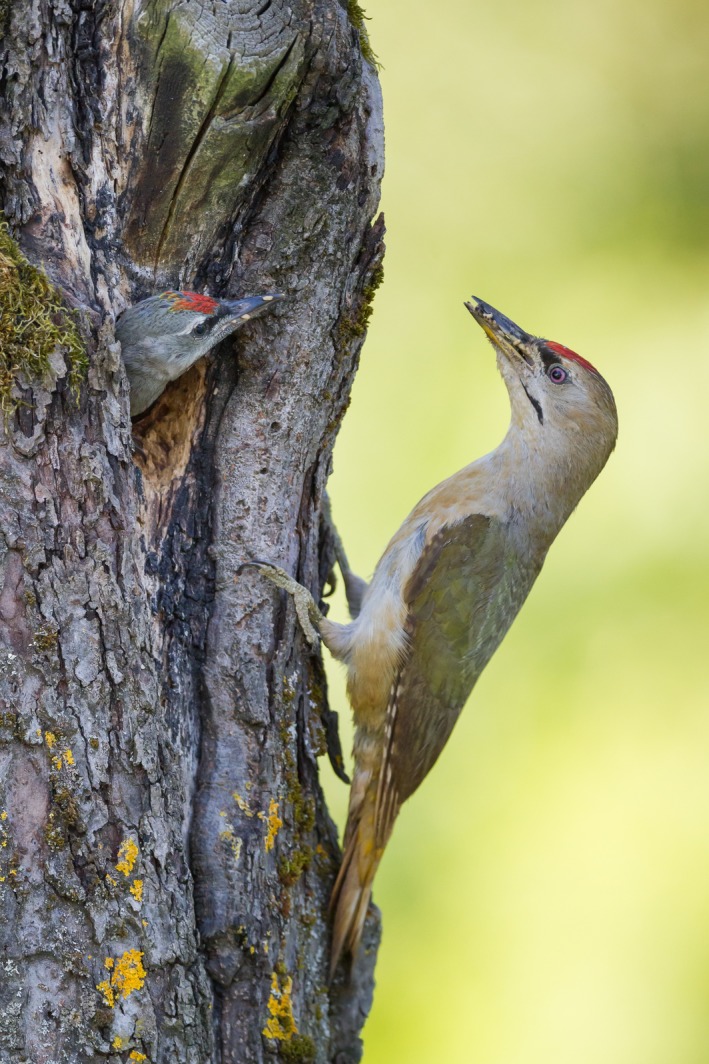
Grey‐headed Woodpecker (*Picus canus* ssp. *canus*), Germany. Image kindly provided by Ralph Martin (http://www.visual-nature.de)

We used Plot Digitizer 2.6.8 (http://plotdigitizer.sourceforge.net/) to extract data that were only provided in figures. In case of missing data, authors were contacted. If only range was provided, the *SD* of the mean DBH was approximated by dividing the range of DBH values by four, equivalent to 95% of the data in a normal distribution (Altman & Bland, 2005). Missing values for the amount of sampled dead and broken‐top trees were approximated by a sample size of 10, which was a conservative value for the median amount of sampled cavity trees across studies. Data were analyzed in R software version 3.2.1 (R Core Team, 2017). Study selection from the literature search results was supported by student assistants. Therefore, the kappa test was used to test the reliability of study inclusion criteria (Cohen, 1960). We drew a sample of 10% of the studies found during the literature search (conducted in 2016) to calculate the kappa coefficient with the “psych” package (Revelle, 2016). The resulting kappa coefficient of 0.65 confirms that the study selection process was different from random study selection (which would have been indicated by a kappa coefficient of zero) and could be reproduced with high agreement between different persons. Effect sizes were computed as log response ratio and log relative risk (Hedges, Gurevitch, & Curtis, 1999; Viechtbauer, 2010). For easier interpretation, back‐transformed effect sizes are presented in the forest plots. In this study, the response ratio was defined as the ratio of the average DBHs of nest and available trees. A response ratio >1 therefore indicates that large‐diameter nest trees were selected for nesting (or, more specifically, a value of 2 indicates that the mean of the selected trees was twice the mean diameter of the available trees). The relative risk was used as a measure of effect size for the binary variables in the data set, which included the vital status (dead/alive) and the tree crown status (broken/intact) of nest and available trees. The relative risk is the ratio of the probability that an event occurs in the treatment group to the probability that the event occurs in the control group (Viechtbauer, 2010). In the context of this study, the values “dead” and “broken” of the respective binary variables were assigned to the treatment group. Therefore, a relative probability of tree selection (relative risk) >1 indicates that trees with the studied characteristics were selected by cavity nesters (Figure 1).

To estimate the effect sizes DBH, vitality, and crown status, we used mixed‐effect models (rma.mv, R package “metafor”, version 1.9‐9, Viechtbauer, 2010) with effect size as the dependent variable and two random effects for bird species and for study. To identify the relative explanatory power of the variables biome, forest type, and naturalness, we computed the Akaike information criterion (AIC, Burnham & Anderson, 2002, pp. 60; Gerstner, Dormann, Stein, Manceur, & Seppelt, 2014) and ΔAIC (the change in the AIC from the models without to with the respective explanatory variable). To spot possible trends depending on these explanatory variables (biome, forest type, and naturalness), we calculated subgroup effect size estimates for all three effect size measures (DBH, vital status, crown status). The probability of selecting a tree for nesting depending on DBH was modeled by a logistic regression model (glmer, R package “lme4,” version 1.1‐10, Bates, Mächler, Bolker, & Walker, 2015). We fitted a generalized additive mixed model (gamm4, R package “gamm4,” version 0.2‐3, Wood & Scheipl, 2014) to show the relationship between the effect size DBH and DBH of available trees. Model assumptions were checked for all models (R package “DHARMa,” version 0.1.5 was used for the logistic regression model, Hartig, 2017) by examining QQ‐plots and residuals vs. predicted values plots. Assumptions were fulfilled acceptably. To assess publication bias, funnel plots were checked for asymmetry with Egger's regression (Egger, Davey Smith, Schneider, & Minder, 1997). The results of Egger's regression indicated that studies reporting significant differences between nest and available trees were indeed more likely to be published (for funnel plots, see Supporting information Figures S4–S6). A list of data sources used in the study is provided in the data sources section. Furthermore, all data are available as Supporting information Data S1.

## 3 RESULTS

Our data set contained 47 cavity‐nesting bird species native to coniferous and deciduous forests in Europe (e.g., great and middle spotted woodpecker, *Dendrocopos major* and *medius*) and North America (e.g., black‐backed woodpecker, *Picoides arcticus*; brown creeper, *Certhia americana*). Fifty‐one studies contributed data to this meta‐analysis. Four studies complemented data of already included studies as they were published by the same authors for the same study area. Most studies (38 of 47) were from the temperate forest biome. Forests dominated by coniferous (25 studies) or deciduous (14 studies) tree species were commonly researched. Mixed forests were underrepresented (eight studies).

Nest trees (*N* = 6473) were on average 13.3 cm thicker than available trees, which had a mean diameter of 35.6 cm. Nests were on average located at a height of 8 m (standard error: 0.5 m). Nonexcavators selected nest cavities that were 97 cm below the overall average height of all cavities in the data set. Quantitative recommendations given in the reviewed studies are shown in Table 1, indicating that across all studies, authors deemed trees with a DBH larger than 20 cm as suitable nest tree.

**Table 1:** Overview of quantitative management recommendations given in the reviewed studies to provide suitable conditions for cavity‐nesting birds. Included were all studies that provided recommendations on potential nest‐tree DBH values

Forest type (location)	Potential nest‐tree DBH (study focus)	Amount of potential nest trees per ha	Source
Coniferous (California)	87% of trees: DBH ≥ 40 cm, mean DBH: ≥70 cm (live and dead trees)	–	Milne and Hejl (1989)
Coniferous (Oregon)	>54 cm, 33% limbs and bark left, slightly decayed (live and dead trees)	–	Bull (1987)
Coniferous (California)	>38 cm, especially white fir (dead trees)	11 soft snags (≥15 years)/ha	Raphael & White (1984)
Coniferous (Oregon)	≥28 cm, hardness 3–4 = 19–125 years after death of tree; stage definitions from Cline, Berg, and Wight (1980), only for clearcuts (dead trees)	≥14 soft snags (≥19 years)/ha with bark cover ≥10%	Schreiber and deCalesta (1992)
Coniferous (Washington)	≥25 cm, for more species: >48 cm (dead trees)	15–35 snags (≥25 cm)/ha	Haggard and Gaines (2001)
Coniferous (Oregon)	>23 cm (dead trees)	–	Wightman et al. (2010)
Coniferous (Idaho)	≥23 cm (dead trees)	≥204 snags (DBH ≥23 cm)/ha	Saab et al. (2009)
Coniferous (Quebec)	>20 cm (dead trees)	Patches of mature/old‐growth burned forest (size: ≥20 ha)	Nappi and Drapeau (2011)
Mixed (Quebec)	≥27 cm for a 50% probability of selection (live and dead trees)	In cutblocks ≤10 live and dead trees/ha with a DBH ≥27 cm (for a 50% probability of selection); in two‐story or irregular forests ≥200 dead trees (with crown and most bark remaining, DBH ≥9 cm)/ha (for a 50% probability of selection)	Tremblay et al. (2015)

**Figure 2:**
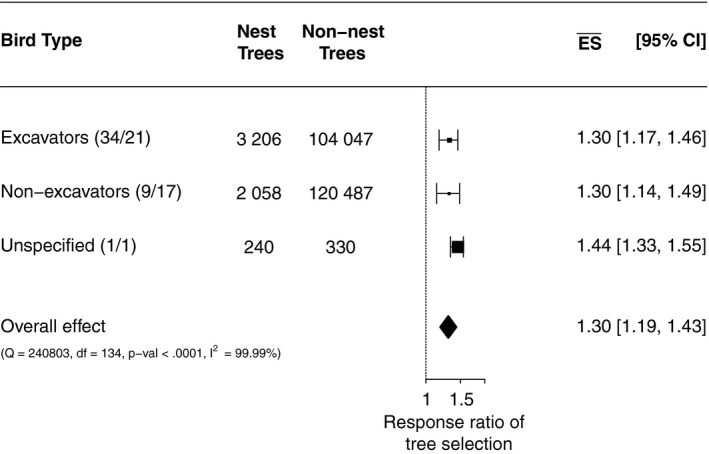
Forest plot showing subgroups and the overall effect of all subgroups combined on log‐scaled *x*‐axis. The vertical line is the line of no effect. The response ratio of tree selection is >1, which indicates that large‐diameter trees were selected for nesting by cavity‐nesting birds. Numbers in parenthesis refer to number of studies/bird species contributing to this category. Unspecified bird species are counted as one single species because only one overall effect size could be estimated for these species

**Figure 3:**
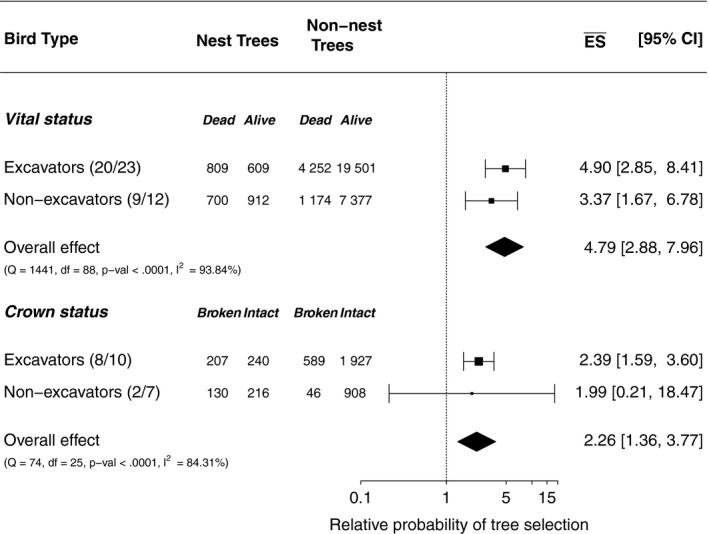
Forest plot showing subgroups for vital status and crown status and the overall effect of all subgroups combined on log‐scaled *x*‐axis. The relative probabilities of tree selection are >1 which indicates that the proportion of trees that were dead or had a broken crown was higher for nest than available trees. Numbers in parenthesis refer to number of studies/bird species contributing to this category

**Figure 4:**
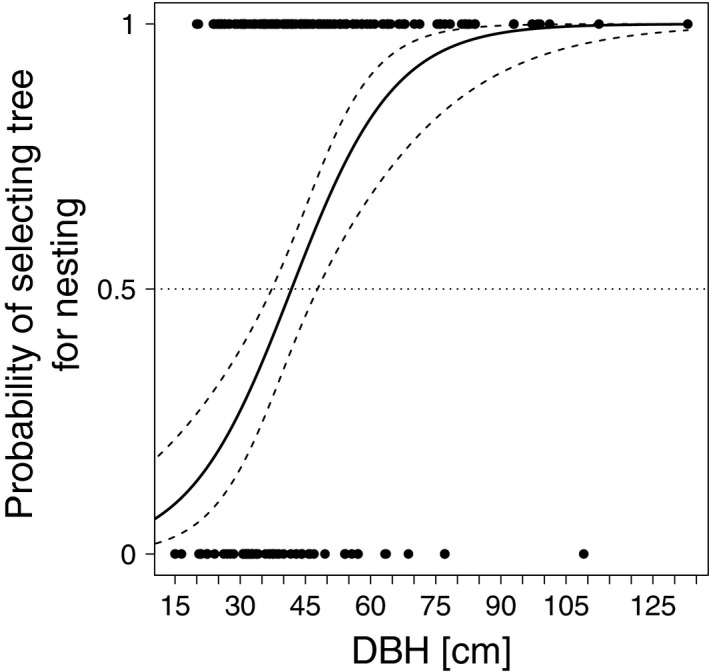
Predicted probability of tree selection by a nest‐site seeking bird, based on 176 paired use‐available‐data for diameter at breast height (DBH) measurements. Below 43 cm DBH trees are discriminated against, above that value they are selected for. Note that this does not describe the probability of a tree having a cavity, but of a bird selecting this tree diameter for nesting (Manly, McDonald, Thomas, McDonald, & Erickson, 2002, pp. 16). Individual studies may have contributed several nest‐tree DBH values for different bird species, forest types (e.g. logged vs. old‐growth) or trees (snags vs. live trees). The curve (solid line, dashed lines indicate 95% confidence intervals for fixed effects) was predicted from a generalized linear mixed model with two random effects for bird species and study

The effect sizes DBH (Figure 2), vital status, and crown status (Figure 3) were >1. This indicates that large‐diameter, dead, and broken‐crown trees were selected by cavity‐nesting birds more often for nesting than the mean of all available trees. For all three effect sizes (DBH, vital status, and crown status), there were only slight differences between nonexcavators/excavators. The probability of nest selection increased as the tree diameter increased (Figure 4). We found that trees with a diameter ≥43 cm had a probability >50% to be selected as a nest tree.

**Figure 5:**
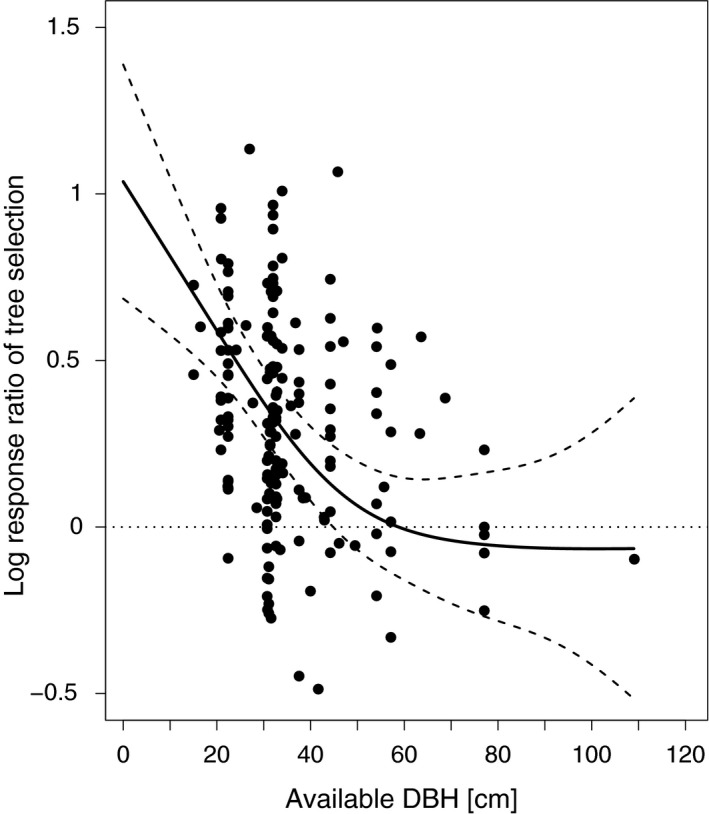
Selection for nest site (log of the response ratio used in Figure 2) as a function of available DBH. The curve (solid line, dashed lines indicate 95% confidence intervals) was predicted from a generalized additive mixed model with two random effects for bird species and study (*N* = 176). A value of 0 indicates no difference between the selected and the available DBH. Values >0 indicate that the mean available DBH was selected against

The effect size of selection decreased as the DBH of available trees increased (Figure 5), suggesting that DBH is an important ecological indicator for cavity‐nesting birds only in forests with low mean DBH values (<45 cm, considering uncertainty) of available trees.

### 3.1 Influence of the explanatory variables biome, forest type, and naturalness

The lowest AIC values were achieved for the effect size DBH by the mixed‐effect model that included naturalness (ΔAIC: 58), and by the model that included forest type for the effect sizes vitality (ΔAIC: 6) and crown status (ΔAIC: 5).

Effect size estimates for the explanatory variables were only slightly different between subgroups (see Supporting information Figure S7). Except for two subgroups (deciduous and mixed forests, both crown status), all estimates were >1. This indicates that large trees, dead trees, and trees with broken crowns were selected by cavity‐nesting birds across biomes, forest types, and degree of naturalness. The minimum DBH values of nest trees for different forest types were 20 cm (coniferous), 30.2 cm (deciduous), and 20.4 cm (mixed). The lowest DBH value of available trees was 15 cm in a temperate forest dominated by coniferous tree species.

## 4 DISCUSSION

### 4.1 The sensitivity of nest‐tree selection to tree characteristics

Our results suggest that trees with a large DBH are preferably selected as nest trees (Figures 2 and 4). In particular for larger excavators (e.g. pileated woodpeckers, Bull, 1987) the nest tree should have a certain minimum DBH for physical reasons in order to sustain a cavity. Therefore, it was expected that large trees would be selected for nesting, which was confirmed by most studies that contributed data (e.g. Bull, 1987; Smith, Warkentin, & Moroni, 2008; Tremblay et al., 2015). However, our results suggest that this is only true when the mean DBH in the forest is low, which might explain that some studies (e.g. Milne & Hejl, 1989) did not find a positive association between DBH and the probability of nest‐tree selection. Indeed, there is evidence from individual studies (e.g. Aubry & Raley, 2002; Seavy et al., 2012), that suggests the importance of DBH as an ecological indicator for cavity‐nesting birds during nest‐tree selection decreases as the DBH of available trees increases (Figure 5). This nonlinear effect of DBH also explains the huge heterogeneity across studies (*I* ^2^‐values larger than 80% in Figures 2 and 3). The power of naturalness to change the AIC value confirms that the variance in the magnitude of the effect size DBH can be attributed substantially to forest structure. Size of cavity‐nesting bird species and tree species also influences the nest‐tree DBH (e.g. Cooke & Hannon, 2012; Saab, Russell, & Dudley, 2009) and therefore the effect size DBH (see Supporting information Appendix S1: Figure S1). For example, Martin et al. (2004) found that all 20 studied cavity‐nesting bird species preferably nested in quaking aspen (*Populus tremuloides*) presumably due to favorable tree characteristics such as soft heartwood and solid sapwood combined with a tendency to remain standing after death (Martin et al., 2004).

Based on our meta‐analysis, dead trees and broken‐crown trees were selected more often for nesting than available live trees and trees with intact crowns. This finding was expected as other studies have argued previously that the tree crown condition is an important ecological indicator for cavity nesters (e.g. Conner, Hooper, Crawford, & Mosby, 1975; Steeger & Hitchcock, 1998). Injuries such as broken crowns (Wagener & Davidson, 1954, p. 68) make trees more susceptible to fungi, which accelerates the decay of heartwood (Wagener & Davidson, 1954, pp. 61). Such infected trees are more easily excavated by cavity nesters and therefore selected for nesting (e.g. Aubry & Raley, 2002; Conner et al., 1975; Steeger & Hitchcock, 1998).

However, previous studies did not invariably support the selection of dead trees or broken‐crown trees by cavity‐nesting birds. For example, excavators (e.g. Hutto & Gallo, 2006; Martin et al., 2004) and nonexcavators (e.g. Dobkin et al., 1995) were observed to nest equally in live and dead trees or even to select live trees. Similarly, the selection of nest trees with broken crowns lacks consistent support in the literature (e.g. Martin et al., 2004; Seavy et al., 2012). The power of forest type to change the AIC value suggests that local conditions explain the inconsistent findings of previous studies. Further, some cavity‐nesting bird species have a greater preference for dead trees in comparison with other cavity nesters (e.g. Hutto & Gallo, 2006; Martin et al., 2004) and therefore influence the magnitude of the effect sizes vital and crown status (see Supporting information Appendix S1: Figures S2 and S3). For example, black‐capped chickadees (*Poecile atricapillus*) and red‐breasted nuthatches (*Sitta canadensis*) were reported to nest more frequently in dead trees than hairy woodpeckers (*Picoides villosus*, Cooke & Hannon, 2012).

### 4.2 Management implications

Overall, the increased statistical power inherent to meta‐analyses indicates that the studied cavity‐nesting birds (see Supporting information Appendix S1: Figures S1–S3 for exceptions) select for trees with larger DBH, dead trees, or trees with broken crowns. The realization of these results through their application in practice or implementation in forest management guidelines is influenced by factors on regional and local scales (e.g., bird species, forest type, naturalness, harvesting technique, legal requirements). Clearly, the retention of large standing dead trees in places that are frequently accessed by forest workers or visitors should be in compliance with existing safety regulations (specified e.g. in ForstBW 2016; Forestry Commission 2017, pp. 50; Humphrey & Bailey, 2012, pp. 15; OMNR 2010, pp. 19). If it is the aim to improve nesting opportunities for small‐sized cavity nesters, it may already be enough to retain small trees as for these birds the retention of large trees has lower importance than for large cavity‐nesting birds (e.g. Cooke & Hannon, 2012). The necessity of retaining large trees for cavity‐nester conservation also decreases as the abundance of large trees increases (Figure 5).

Our aim was to clarify general patterns in nest‐tree selection as this is a strength of meta‐analytical techniques (CEE 2013, Koricheva et al., 2013, p. 8, table 1.1). Inference to make more specific management recommendations, however, is determined by the available studies which were mostly conducted in the temperate biome. Nevertheless, this study has important implications across harvesting systems that employ tree retention.

Retention forestry uses selected forest elements such as large, living, and dead trees (Gustafsson et al., 2012) which are retained during harvest with the aim to enhance forest connectivity, continuity, and structure (Franklin, Berg, Thornburgh, & Tappeiner, 1997). Besides being already implemented in rotation forest management, retention forestry can also be practiced in continuous cover forestry (e.g. selection systems; Franklin et al., 1997; Bauhus, Puettmann, & Messier, 2009; Gustafsson et al., 2012). In current selection systems (now practiced globally, Schütz, Pukkala, Donoso, & von Gadow, 2012, pp. 5) the focus is on retaining single (or small groups of) large trees with particular features (e.g., dead trees, trees with broken crowns) that would otherwise be felled (Bauhus et al., 2009; ForstBW, 2016). The effects of retaining structural elements in forests under selection harvest techniques (e.g., single tree selection) have rarely been meta‐analyzed (Fedrowitz et al., 2014; Lassauce, Paillet, Jactel, & Bouget, 2011; Mori & Kitagawa, 2014; Prevedello, Almeida‐Gomes, & Lindenmayer, 2018; Rosenvald & Lõhmus, 2008; Seibold et al., 2015). Arguably, the retention of large trees may be far less influential in such systems. Earlier findings (Fedrowitz et al., 2014; Mori & Kitagawa, 2014; Rosenvald & Lõhmus, 2008) about biodiversity enhancing effects of retention forestry are supported by the results of this meta‐analysis. Our study expands on this as it indicates for the first time the importance of biological legacies (Franklin et al., 1997) in selection systems.

Arguments have been made to include this knowledge in policies for the conservation of ecologically important forest structures (Lindenmayer et al., 2014). Major forest management guides in different countries (Canada, OMNR 2010; UK, Forestry Commission 2017) now mention the retention of such key structures. In some federal states of Germany (e.g., Baden‐Württemberg and Bavaria), regulations are in place that aim to provide habitats for species depending on old‐growth forest structures (ForstBW, 2016; Nüßlein & Becher, 2015; Spielmann, Bücking, Quadt, & Krumm, 2013, pp. 33). Our quantitative results with regard to the size and condition of biological legacies confirm the approach of maintaining and promoting old‐growth forest structures.

## 5 CONCLUSIONS FOR PRACTICE

This meta‐analysis shows the importance of big and decaying trees for cavity‐nesting birds. In harvesting systems with tree retention such as they are now common in both Europe and North America, larger trees should be retained if the aim is to increase abundance of many different cavity‐nesting birds. Target tree sizes depend on cavity‐nesting bird species and forest naturalness and also need to consider safety regulations and distance to infrastructure (e.g., walking tracks, skid trails). DBH is only one of several proxies that can be useful in forest management. Further, tree characteristics such as the crown status or vital status also have predictive power and should be considered during the selection of suitable trees for retention. Retaining such trees (instead of only focussing on trees with large DBH) might also be more realistic if economic factors are taken into consideration.

## DATA SOURCES

Studies included in the meta‐analysis based on the study inclusion criteria (shown in Supporting information Appendix S1: List S1): Raphael & White (1984); Zarnowitz and Manuwal (1985); Bull (1987); Gutzwiller and Anderson (1987); O'Halloran and Conner (1987); Harestad and Keisker (1989); Milne and Hejl (1989); Renken and Wiggers (1989); Li and Martin (1991); Schreiber and deCalesta (1992); Conway and Martin (1993); Hogstad and Stenberg (1994); Dobkin et al. (1995); Stenberg (1996); Smith (1997); Vierling (1997); Linder and Anderson (1998); Steeger and Hitchcock (1998); Haggard and Gaines (2001); Aubry and Raley (2002); Buchanan, Rogers, Pierce, and Jacobson (2003); Dornak et al. (2004); Hartwig, Eastman, and Harestad (2004); Martin et al. (2004); Kosiński and Winiecki (2004); Walton, Ortega, and Ortega (2005); Hutto and Gallo (2006); Kosiński, Ksit, and Winiecki (2006); Vierling and Lentile (2006); King, Brashear, and Reiman (2007); Mahon, Martin, and Steventon (2007); Pasinelli (2007); Walankiewicz, Czeszczewik, and Mitrus (2007); Bagne, Purcell, and Rotenberry (2008); Cornelius (2008); Gentry and Vierling (2008); Bonnot, Millspaugh, and Rumble (2009); Saab et al. (2009); Vierling, Gentry, and Haines (2009); Wightman, Saab, Forristal, Mellen‐McLean, and Markus (2010); Nappi and Drapeau (2011); Seavy et al. (2012); Hudson and Bollinger (2013); Shiina, Hasegawa, and Higashi (2013); Cikovic, Barišic, Tutiš, and Kralj (2014); Berl, Edwards, and Bolsinger (2015); Lorenz et al. (2015); Tremblay et al. (2015); Geleynse et al. (2016); Latif, Saab, Hollenbeck, and Dudley (2016); Ónodi and Winkler (2016).

## AUTHORS’ CONTRIBUTIONS

CFD and FG contributed first ideas; FG collected the data and provided the first draft manuscript; CFD and FG analyzed the data. Both authors contributed substantially to the writing and gave final approval for publication.
