# Supplementary material for: Decaying trees improve nesting opportunities for cavity‐nesting birds in temperate and boreal forests: A meta‐analysis and implications for retention forestry
Source: Ecol Evol. 2018 Jul 16;8(16):8616–26. doi: 10.1002/ece3.4245 (PMC6144968; doi:10.1002/ece3.4245)
Supplement: Supplementary file 1 [file ECE3-8-8616-s001.pdf]

Supporting Information for: "Decaying trees improve nesting opportunities for cavity-nesting birds in temperate and boreal forests: A meta-analysis and implications for retention forestry"

List S1: Study Inclusion Criteria sorted by relevance:

(1) Studies that provided information about the amount of nest and available trees with broken/intact crown were included.

(2) Studies that provided information about the amount of nest and available trees that were dead/alive were included.

(3) Studies that reported the diameter at breast height (DBH) of trees that hosted an active cavity and random trees as numerical data (e.g. mean DBH) in the text/supplementary data/appendix or a figure were included.

  (3a) Studies that reported DBH measurements taken from trees with active *and* inactive nests were included in the literature review.

  (3b) Studies that included nest trees in the "random" tree DBH value were included if less than 25% of "random" trees were nest trees.

  (3c) Studies were included only if DBH data for non-nest trees were measured on trees that could be considered as being selected randomly (i.e. not based on several selection criteria such as presence of cavities or stick nests, minimum cavity entrance) from the available trees.

(4) Studies about primary (excavators, e.g. white-headed woodpecker, *Picoides albolarvatus*) cavity-nesting birds were included.

(5) Studies about secondary (non-excavators, e.g. mountain chickadee, *Poecile gambeli*) cavity-nesting birds were included.

(6) Studies carried out in forests of the boreal and temperate regions were included (Def. boreal and temperate region based on study area description and complemented by the World Biomes Map, `http://www.worldbiomes.com/biomes_map.htm`).

(7) Only studies published in peer-reviewed journals were included.

(8) If it was stated that trees hosting different nest types (e.g. top cavities, platforms, stick nests) were studied information to distinguish from side-cavities had to be included. If just one nest-tree DBH was reported for different nest types the study was included if more than 90% of total cavities were side-cavities.

(9) We included observational studies (Level of Evidence 3 of the evidence hierarchy, Mupepele *et al.* 2016) if data measured in the field were reported that also fulfilled our other inclusion criteria.

Table S1: Search strings used for the meta-analysis. In November 2017 the search was updated with search string two.

Database	Search String
1. WoS	TS=((“avian” OR “avifauna” OR “aves” OR “ornithology” OR “bird” OR “woodpecker” OR “chickadee” OR “picidae” OR “poecile” OR “picoides” OR “strigidae” OR “strigiforme” OR “cavity nester” OR “nest” OR “excavator”) AND (“habitat requirement” OR “old-growth” OR “snag”) AND (“dbh” OR “threshold”)) Refined by: Research Areas: (Environmental Sciences Ecology OR Forestry OR Biodiversity Conservation OR Zoology OR Reproductive Biology OR Evolutionary Biology OR Developmental Biology)
2. WoS	TS=(“woodpecker” AND “nest”) Refined by: Document Types: (article OR review OR abstract) and Research Areas: (Environmental Sciences Ecology OR Forestry OR Biodiversity Conservation OR Zoology OR Reproductive Biology OR Evolutionary Biology OR Developmental Biology)
3. GS	((avian OR avifauna OR aves OR bird OR woodpecker OR picidae OR poecile OR cavity nester OR nest OR excavator) AND (habitat-requirement OR old-growth OR snag OR silviculture) AND (dbh OR basal OR threshold))
4. Cab	((avian or avifauna or aves or ornithology or bird or woodpecker or chickadee or picidae or poecile or picoides or strigidae or strigiforme or cavity nester or nest or excavator) and (habitat requirement or old growth or snag or silviculture) and (dbh or height or basal or density or threshold)) .mp. [mp=abstract, author, book author, book title, corporate author, collection authors, collection title, corporate author word, heading word, subject heading, title, year]
5. GeoRef	((avian OR avifauna OR aves OR ornithology OR bird OR woodpecker OR chickadee OR picidae OR poecile OR cavity nester OR nest OR excavator OR picoides OR strigidae OR strigiforme) AND (habitat requirement OR old growth OR snag OR silviculture) AND (dbh OR height OR basal OR density OR threshold))
6. DOAJ	((avian OR avifauna OR aves OR ornithology OR bird OR woodpecker OR chickadee OR picidae OR poecile OR cavity nester OR nest OR excavator OR picoides OR strigidae OR strigiforme) AND (habitat requirement OR old growth OR snag OR silviculture) AND (dbh OR height OR basal OR density OR threshold))

Database	Search String
7. BioOne	((“avian” OR “avifauna” OR “aves” OR “ornithology” OR “bird” OR “woodpecker” OR “chickadee” OR “picidae” OR “poecile” OR “picoides” OR “strigidae” OR “strigiforme” OR “cavity nester” OR “nest” OR “excavator”) AND (“habitat requirement” OR “old-growth” OR “snag” OR “silviculture”) AND (“dbh” OR “height” OR “basal” OR “density” OR “threshold”))
8. Springer	((“avian” OR “avifauna” OR “aves” OR “ornithology” OR “bird” OR “woodpecker” OR “chickadee” OR “picidae” OR “poecile” OR “picoides” OR “strigidae” OR “strigiforme” OR “cavity nester” OR “nest” OR “excavator”) AND (“habitat requirement” OR “old-growth” OR “snag” OR “silviculture”) AND (“dbh” OR “height” OR “basal” OR “density” OR “threshold”)) within Forestry AND Ecology
9. ScienceDirect	tak((“avian” OR “avifauna” OR “aves” OR “ornithology” OR “bird” OR “woodpecker” OR “chickadee” OR “picidae” OR “poecile” OR “picoides” OR “strigidae” OR “strigiforme” OR “cavity nester” OR “nest” OR “excavator”) AND (“habitat requirement” OR “old-growth” OR “snag” OR “silviculture”) AND (“dbh” OR “height” OR “basal” OR “density” OR “threshold”)
10. JSTOR	((“avian” OR “avifauna” OR “aves” OR “bird” OR “woodpecker” OR “picidae” OR “poecile” OR “cavity nester” OR “nest” OR “excavator”) AND (“habitat requirement” OR “old-growth” OR “snag” OR “silviculture”) AND (“dbh” OR “basal” OR “threshold”)) AND disc:(biologicalsciences-discipline OR ecology-discipline OR botany-discipline OR environmentalscience-discipline OR zoology-discipline)

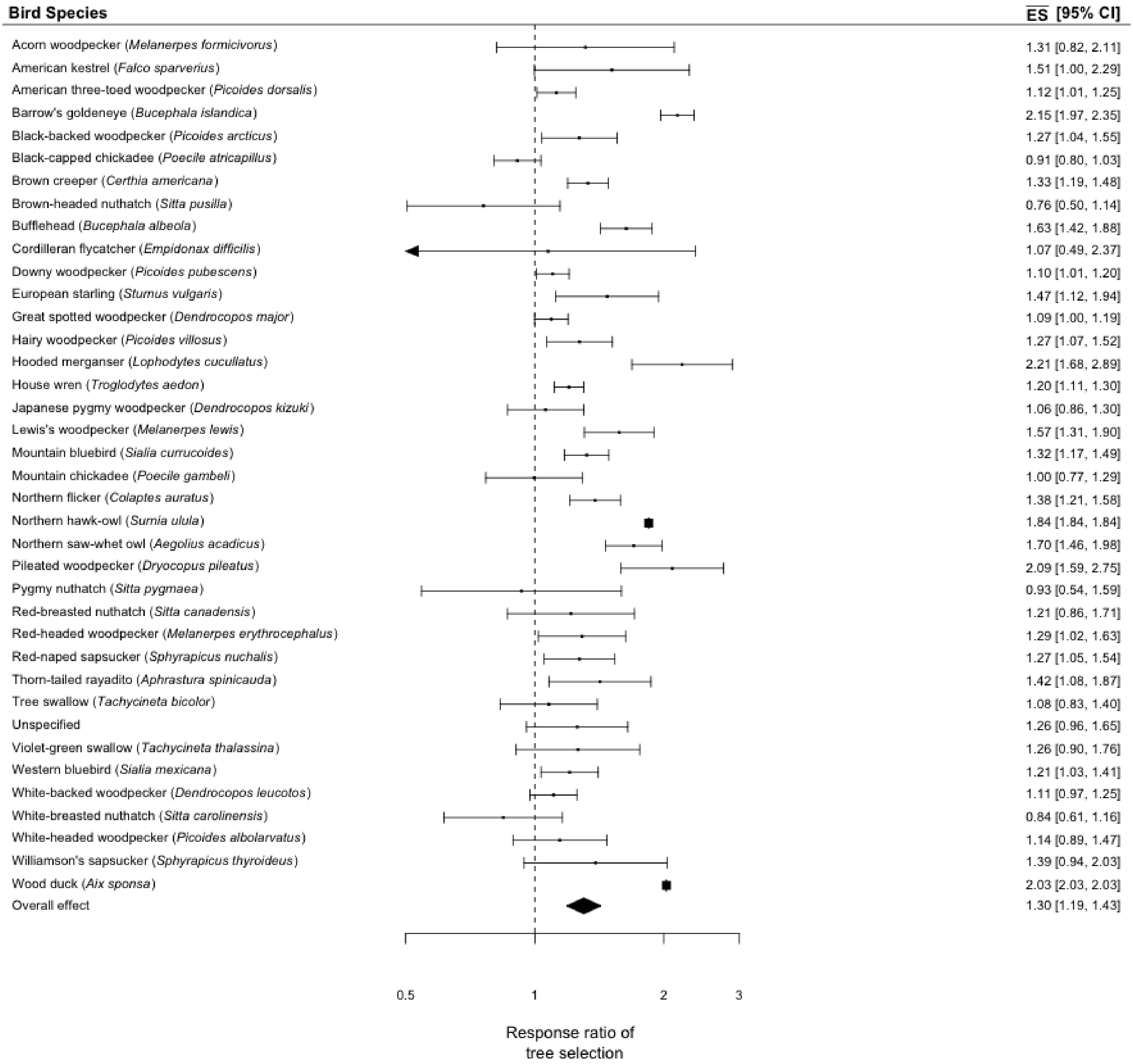

Figure S1: Forest plot for effect size DBH on log-scaled x-axis. The vertical line is the line of no effect. A response ratio >1 indicates that large-diameter trees were preferred for nesting by cavity-nesting birds. The figure indicates that most bird species selected for large nest trees.

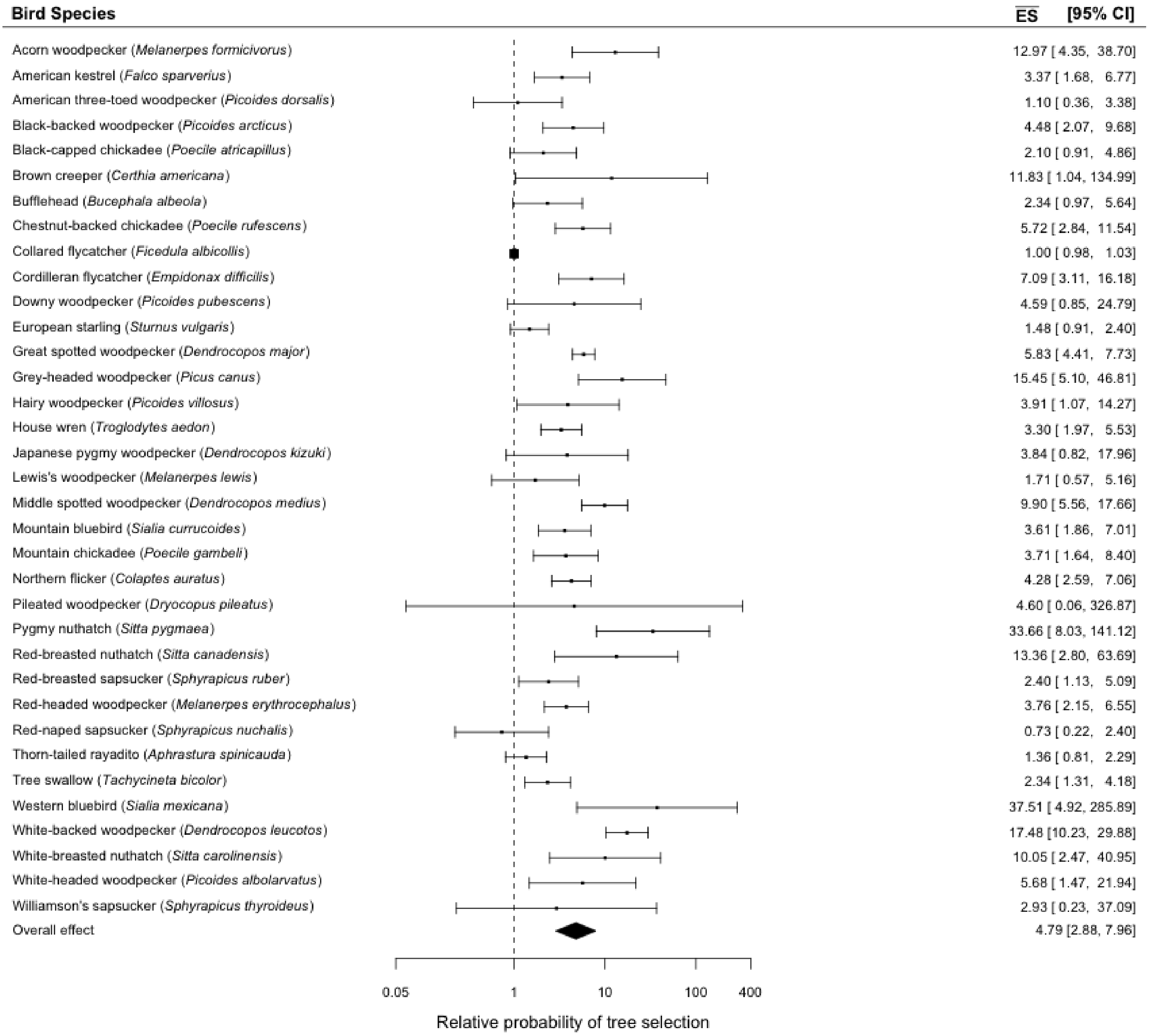

Figure S2: Forest plot for effect size vital status (dead/living tree) on log-scaled x-axis. The vertical line is the line of no effect. Relative probabilities >1 indicate that the probability of being selected as nest tree was higher for dead trees than for live trees. The figure indicates that most bird species selected for dead nest trees.

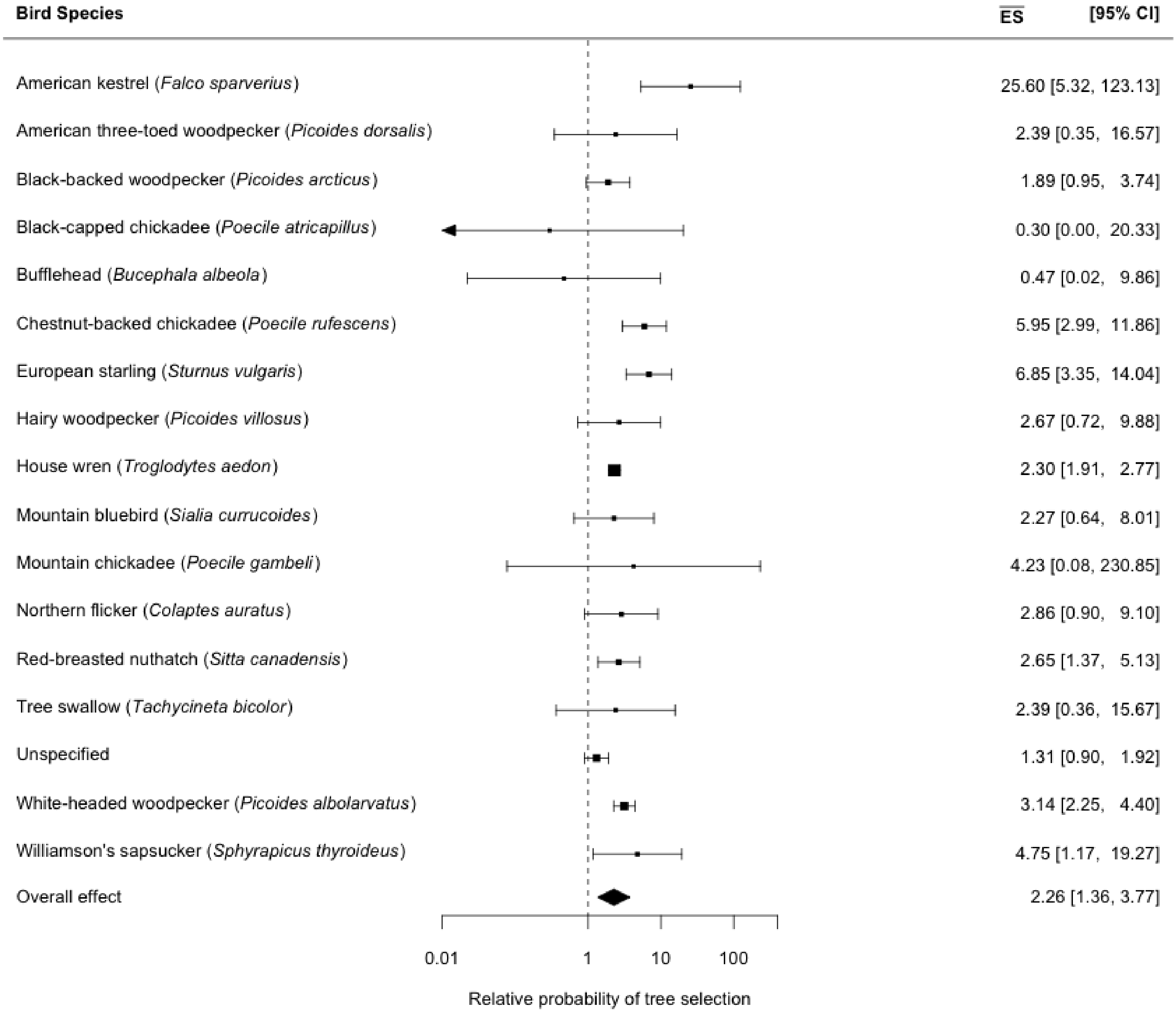

Figure S3: Forest plot for effect size crown status (broken/unbroken crown) on log-scaled x-axis. The vertical line is the line of no effect. Relative probabilities >1 indicate that the probability of being selected as nest tree was higher for broken-crown trees than for intact-crown trees. The figure indicates that most bird species selected for broken-crown nest trees.

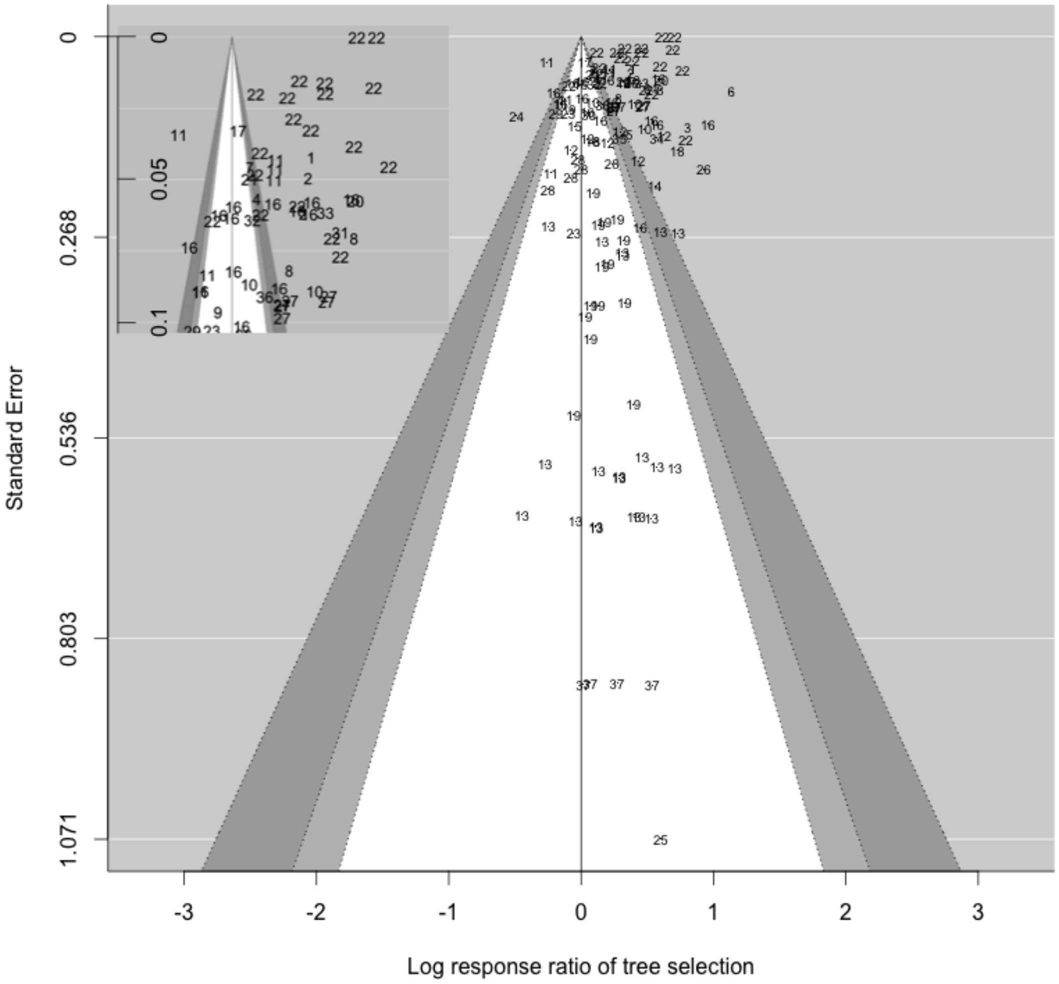

Figure S4: Contour-enhanced funnel plot for effect size DBH. To indicate non-independence same numbers were used if several effect sizes were derived from the same study. In the top-left corner a magnification of clustered effect sizes is provided. Shading indicates the p-value (white: >.10, grey: from .10 to .05, dark grey: from .05 to .01, area outside the triangle: <.01). Intercept of Egger's regression indicated asymmetry (publication bias): a = 0.27 (p-value: <0.001). The figure indicates that publication bias was present for the effect size DBH. Please note that funnel plots do not account for the multi-level structure of our data and Egger's regression may therefore detect publication bias that in fact is accounted for in our mixed-model analysis (Egger *et al.* 1997; Koricheva *et al.* 2013, pp. 218).

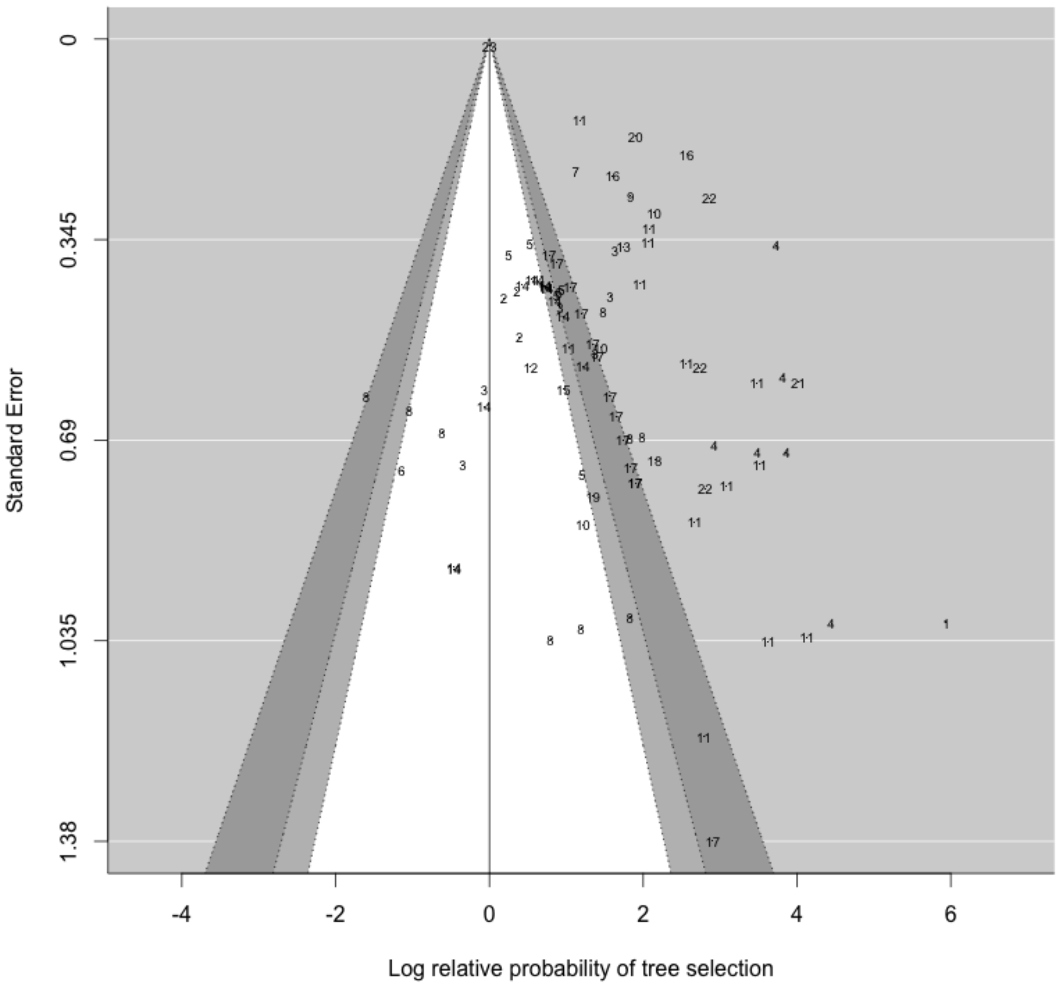

Figure S5: Contour-enhanced funnel plot for effect size vital status. To indicate non-independence same numbers were used if several effect sizes were derived from the same study. Shading indicates the p-value (white: >.10, grey: from .10 to .05, dark grey: from .05 to .01, area outside the triangle: <.01). Intercept of Egger's regression indicated asymmetry (publication bias): a = 1.34 (p-value <0.001). The figure indicates that publication bias was present for the effect size DBH. Please note that funnel plots do not account for the multi-level structure of our data and Egger's regression may therefore detect publication bias that in fact is accounted for in our mixed-model analysis (Egger *et al.* 1997; Koricheva *et al.* 2013, pp. 218).

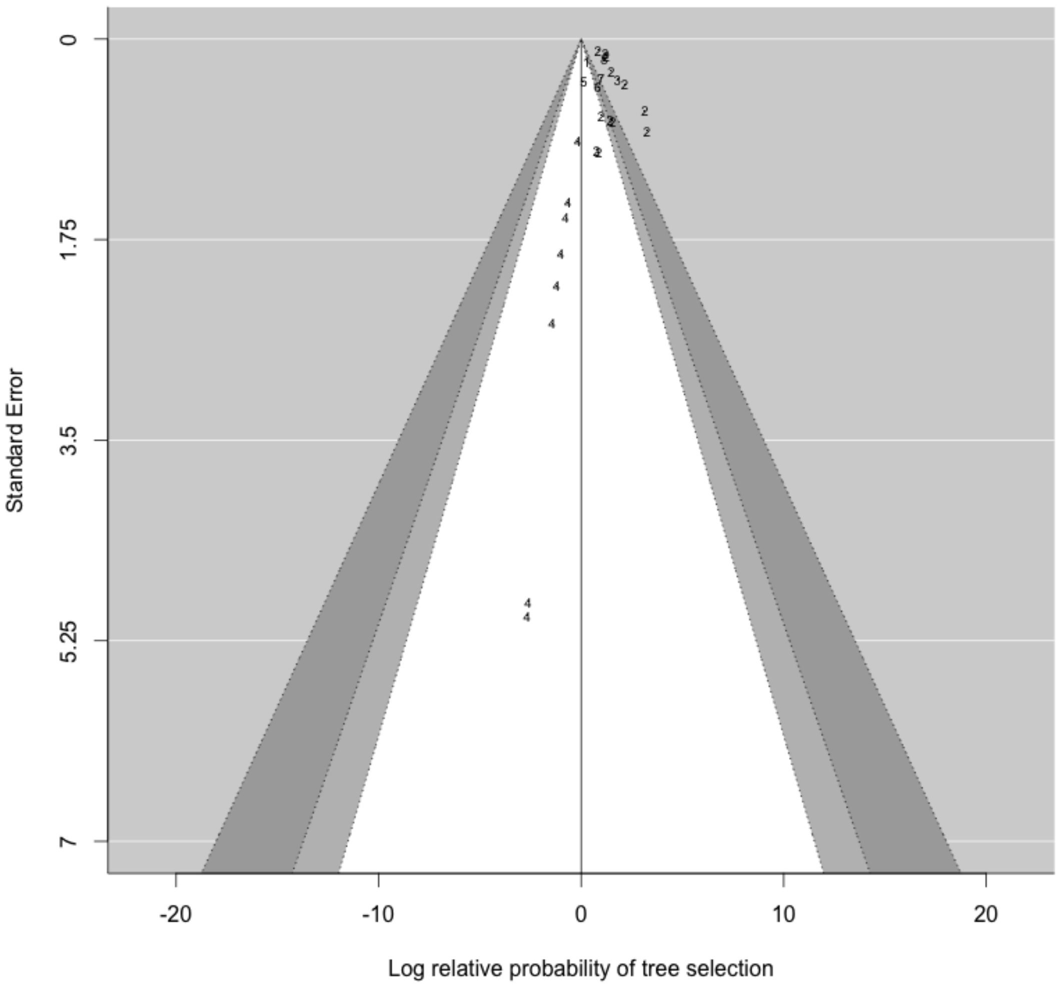

Figure S6: Contour-enhanced funnel plot for effect size crown status. To indicate non-independence same numbers were used if several effect sizes were derived from the same study. Shading indicates the p-value (white: >.10, grey: from .10 to .05, dark grey: from .05 to .01, area outside the triangle: <.01). Intercept of Egger's regression indicated asymmetry (publication bias): a = 0.91 (p-value: <0.001). The figure indicates that publication bias was present for the effect size DBH. Please note that funnel plots do not account for the multi-level structure of our data and Egger's regression may therefore detect publication bias that in fact is accounted for in our mixed-model analysis (Egger *et al.* 1997; Koricheva *et al.* 2013, pp. 218).

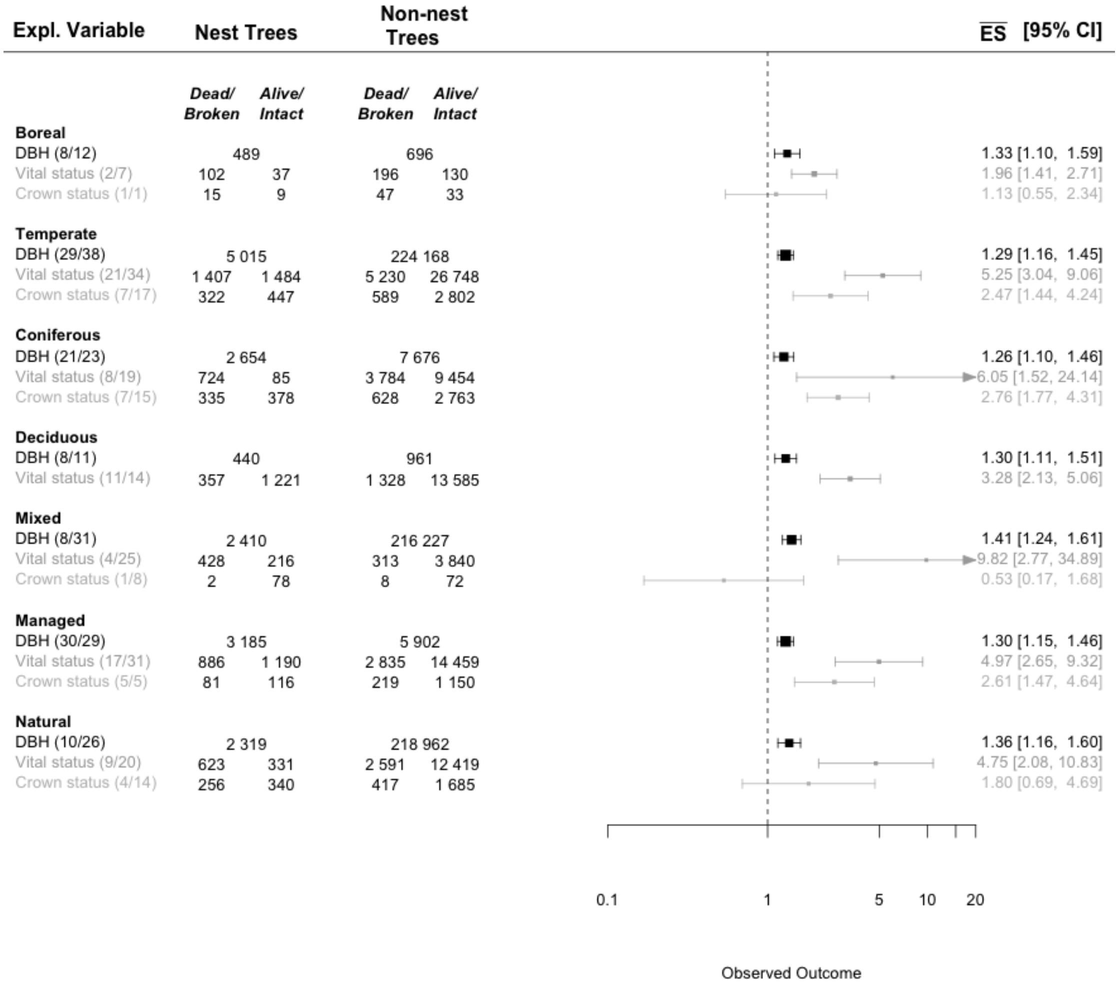

Figure S7: Forest plot showing subgroups of all three effect sizes (DBH, vital status, crown status) for explanatory variables (biom, forest type, naturalness) on log-scaled x-axis. The vertical line is the line of no effect. Effect sizes are only slightly different between subgroups for all three effect sizes. This indicates that large-diameter trees, dead trees and broken-crown trees were preferred for nesting by cavity-nesting birds across bioms, forest types and management regimes. Numbers in parenthesis refer to number of studies/bird species contributing to this category. Unspecified bird species are counted as one single species because only one overall effect size could be estimated for these species.

Authors who provided data

Besides contacting authors of studies to which we had no access we also requested data. In several cases these data were no longer available. This was not the case for the study of Renken & Wiggers (1989). We appreciate very much the efforts made by Ms Rochelle Renken to locate and sending us data.

References

Egger, M., Davey Smith, G., Schneider, M. & Minder, C. (1997) Bias in meta-analysis detected by a simple, graphical test. *British Medical Journal*, **315**, 629–634.

Koricheva, J., Gurevitch, J. & Mengersen, K.L. (2013) *Handbook of Meta-Analysis in Ecology and Evolution.* Princeton University Press, Princeton.

Mupepele, A.C., Walsh, J.C., Sutherland, W.J. & Dormann, C.F. (2016) An evidence assessment tool for ecosystem services and conservation studies. *Ecological Applications*, **26**, 1295–1301.

Renken, R.B. & Wiggers, E.P. (1989) Forest characteristics related to pileated woodpecker territory size in Missouri. *The Condor*, **91**, 642–652.
